# Population genetic analysis in old Montenegrin vineyards reveals ancient ways currently active to generate diversity in *Vitis vinifera*

**DOI:** 10.1038/s41598-020-71918-7

**Published:** 2020-09-14

**Authors:** Vesna Maraš, Javier Tello, Anita Gazivoda, Milena Mugoša, Mirko Perišić, Jovana Raičević, Nataša Štajner, Rafael Ocete, Vladan Božović, Tatjana Popović, Enrique García-Escudero, Miodrag Grbić, José Miguel Martínez-Zapater, Javier Ibáñez

**Affiliations:** 1 13 Jul Plantaže, Radomira Ivanovića br. 2, 8100 Podgorica, Montenegro; 2 Instituto de Ciencias de la Vid y del Vino (CSIC, UR, Gobierno de La Rioja), Ctra. de Burgos Km. 6, 26007 Logroño, Spain; 3 Biotechnical Faculty, Agronomy Department, University of Ljubljana, Jamnikarjeva 101, 1000 Ljubljana, Slovenia; 4 Laboratorio de Entomología Aplicada, Facultad de Biología, Universidad de Sevilla, Avenida de la Reina Mercedes s/n, 41012 Seville, Spain; 5 Faculty for Food Technology, Food Safety and Ecology, University of Donja Gorica, 81000 Donja Gorica, Podgorica, Montenegro; 6 Biotechnical Faculty, University of Montenegro, Mihaila Lalica 1, 81000 Podgorica, Montenegro; 7 Department of Biology, University of Western Ontario, 1151 Richmond Street, London, N6A5B7 Canada; 8 Faculty of Biology, University of Belgrade, Studentski trg. 16, Beograd, 11000 Serbia

**Keywords:** Biodiversity, Agricultural genetics

## Abstract

Global viticulture has evolved following market trends, causing loss of cultivar diversity and traditional practices. In Montenegro, modern viticulture co-exists with a traditional viticulture that still maintains ancient practices and exploits local cultivars. As a result, this region provides a unique opportunity to explore processes increasing genetic diversity. To evaluate the diversity of Montenegrin grapevines and the processes involved in their diversification, we collected and analyzed 419 samples in situ across the country (cultivated plants from old orchards and vines growing in the wild), and 57 local varieties preserved in a grapevine collection. We obtained 144 different genetic profiles, more than 100 corresponding to cultivated grapevines, representing a surprising diversity for one of the smallest European countries. Part of this high diversity reflects historical records indicating multiple and intense introduction events from diverse viticultural regions at different times. Another important gene pool includes many autochthonous varieties, some on the edge of extinction, linked in a complex parentage network where two varieties (Razaklija and Kratošija) played a leading role on the generation of indigenous varieties. Finally, analyses of genetic structure unveiled several putative proto-varieties, likely representing the first steps involved in the generation of new cultivars or even secondary domestication events.

## Introduction

The cultivated grapevine (*Vitis vinifera* subsp. *sativa* L.) was likely first domesticated from the wild grapevine (*V. vinifera* subsp. *sylvestris* (C.C.Gmel.) Hegi) in the Transcaucasian region (modern Georgia, Armenia, and Azerbaijan) about 8,000 years ago^1^. Then, early cultivars spread around the Mediterranean basin following the main human migration routes during several thousand years^2^. During this process, certain vines were selected for their adaptation to regional conditions and their ability to overcome local biotic and abiotic stresses. These vines become stable varieties through their vegetative propagation and evolved through combining mutation and sexual reproduction events with other early-domesticated cultivars or with wild autochthonous vines^2^.

From ancient times to modern days, the Western Balkans have been home of important European civilizations. Illyrians, Greeks, Romans, Byzantines, Ottomans and Austro-Hungarians settled along this region, promoting the exchange of people, commodities and knowledge within close and distant regions (Near Asia, Dalmatia, Magna Graecia, etc.)^3^. First grapevines were introduced in the Balkan Peninsula from the East^4^, with the earliest-known evidences of winemaking found in Northern Greece dating back to ca. 4,300 BCE^5^. Ancient Greeks gradually spread grape varieties across the Western side of the Balkans and the Adriatic islands following main trading routes^2,6^, and Illyrians and other native populations progressively replaced the consumption of cereal-based beverages (mead, beer) with wine^7^. Winemaking and wine trading were two important activities for local economies at that time, as inferred from relics like those found in the necropolis of the coastal city of Budva (in modern Montenegro, dated from the fourth century BCE)^8^. Viticultural practices were intensified by Romans after the Illyrian Wars, as observed in Dionysian/Bacchic iconography, and in many remnants from domestic utensils used for wine transportation or consumption (pottery, amphorae, cups)^7^. Wine production continued during the Byzantine period, and documents like the Medieval Statute of Budva reflects the importance of grapevine cultivation in Montenegro in the Middle Ages. The Statute, dated between 1,426 and 1,442, includes the relevance of certain grapevine varieties for local wine production^9,10^, like the cultivar “Cratosia”, probably referring to the grape cultivar currently known as Kratošija (meaning short-neck in Montenegrin)^9^. Later, many vineyards were destroyed during the Ottoman period, and only some survived in remote regions in the center of the country and in several parts of the coastal regions controlled by Venice^6^. Winemaking practices were partially restored under the rule of Nikola I (1860–1918), which promoted wine exports to Western countries. After several millennia of genetic diversification, most of the grapevine genetic diversity generated in the Balkans was destroyed by the effect of phylloxera and mildews introduced from North America at the end of the nineteenth century^2,11^. As a result, many old autochthonous cultivars of the Western Balkans exist now as isolated plants or as relict populations, often represented by few specimens found in old, traditional vineyards or maintained in ex situ* Vitis* collections^6,12, 13^.

Montenegro played a major role in the long history of grape cultivation in the Western Balkans. Its highly diverse climate conditions, isolated valleys, soil types and orography create highly diverse environments that, together with its historical and geographic context, promoted the generation of a rich grapevine biodiversity^14,15^. Overall, grapes are grown in Montenegro over 2.800 ha, with a gross production of 22.200 t in 2017^16^. Winemaking in Montenegro mainly relies on the production of red wine from two autochthonous grape cultivars, Vranac and Kratošija^17^. Kratošija was the dominant variety in the region until the phylloxera crisis, which forced the removal of many old vines and their replacement by new plant material^11^. In many cases, withered Kratošija vines were replaced by grafted Vranac plants, preferred by growers as it produces highly deep-colored wines^17^. As a result, the Kratošija cultivated area decreased, while Vranac became the most commonly grown and the most emblematic cultivar in Montenegro during the twentieth century^14^. Besides these two major cultivars, other less-known indigenous cultivars can be found in Montenegro and neighboring countries, including Bioka, Čubrica, Krstač and Žižak^6,14,18^.

Worldwide wine consumption patterns have changed radically and rapidly in recent years, influencing changes in viticulture. For a long time, grape growers selected and maintained local grape varieties to fit their viticultural practices and local climates, producing grapes that reached an appropriate concentration of sugars, acids and other compounds to make traditional wines^2^. On the contrary, recent market globalization has caused a dramatic erosion in the diversity of wine grapes planted across the world, moving towards the cultivation of a reduced set of “international varieties”^19^. Thus, the exploration and analysis of winemaking regions where old vineyards are still maintained under traditional management practices can help to understand how European viticulture developed over time before the current standardization. As exemplified in multiple regions^13,18,20,21^, wide surveys in traditional winemaking areas allow the identification of old genetic resources on the edge of extinction for their preservation and eventual exploitation. Grapes from unique native varieties can produce highly distinctive wines with real potential to revitalise local wine industries^18,20,22^, and there is a renewed global interest in local wine varieties from Old World nations to stand out in today highly standardized market. Furthermore, the analysis of these indigenous and traditional varieties has helped to successfully resolve previous doubts about cultivars parentage^23–25^, as well as to shed light on the likely origin and dissemination patterns of certain grapevine cultivars in traditional viticulture regions^20,26^.

The use of nuclear DNA markers, mainly microsatellites (or Simple Sequence Repeats, SSRs) and Single Nucleotide Polymorphisms (SNPs) have contributed decisively to the identification of grapevine varieties^27,28^. Grape varieties are maintained through vegetative propagation, and thus all the plants of any variety present the same genotype for the molecular markers used^29,30^, while a few molecular markers are enough to distinguish any two varieties^29,31^. This fact has allowed to confirm and discover many local and international synonyms (different names for one variety) and homonyms (same name for different varieties)^32,33^. These DNA markers have also been commonly used for determining the existence of genetic structure in sets of vines at local and wider levels^34, 35^. Finally, a few chloroplast DNA markers are informative enough to establish chlorotypes in *Vitis vinifera*, existing only four different major types^36^ that are maternally inherited. Chlorotypes have been useful to determine the female parent in a pedigree, and to show the multiple origins of cultivated grapevine by analyzing the distribution of chlorotypes in local sylvestris and cultivated vines^37,38^.

Here, we present the results of a nation-wide survey of endangered genetic resources in Montenegro, including cultivated plants (human-cared) from old orchards and vines growing in the wild. Samples were characterized by SNP and SSR markers, and unique genetic profiles were compared with international databases for proper varietal identification and for the detection of synonymies and homonymies. In addition, we analyzed Montenegrin grapevine genetic diversity and structure, and unveiled their genetic relationships. These results revealed (1) the origin of modern Montenegrin cultivars from autochthonous and/or introduced varieties, (2) the existence of anachronic proto-varieties in this region, and (3) introgression events from wild vine populations which shape the genetic structure of domesticated grapevines. This work represents a first step toward promoting wider use of indigenous and traditional grapevine varieties in the modern Montenegrin wine industry. It will contribute to the diversification and enrichment of the European wine sector.

## Results

### Sampling and genetic identification of cultivated and wild grapevines in Montenegro

**Figure 1:**
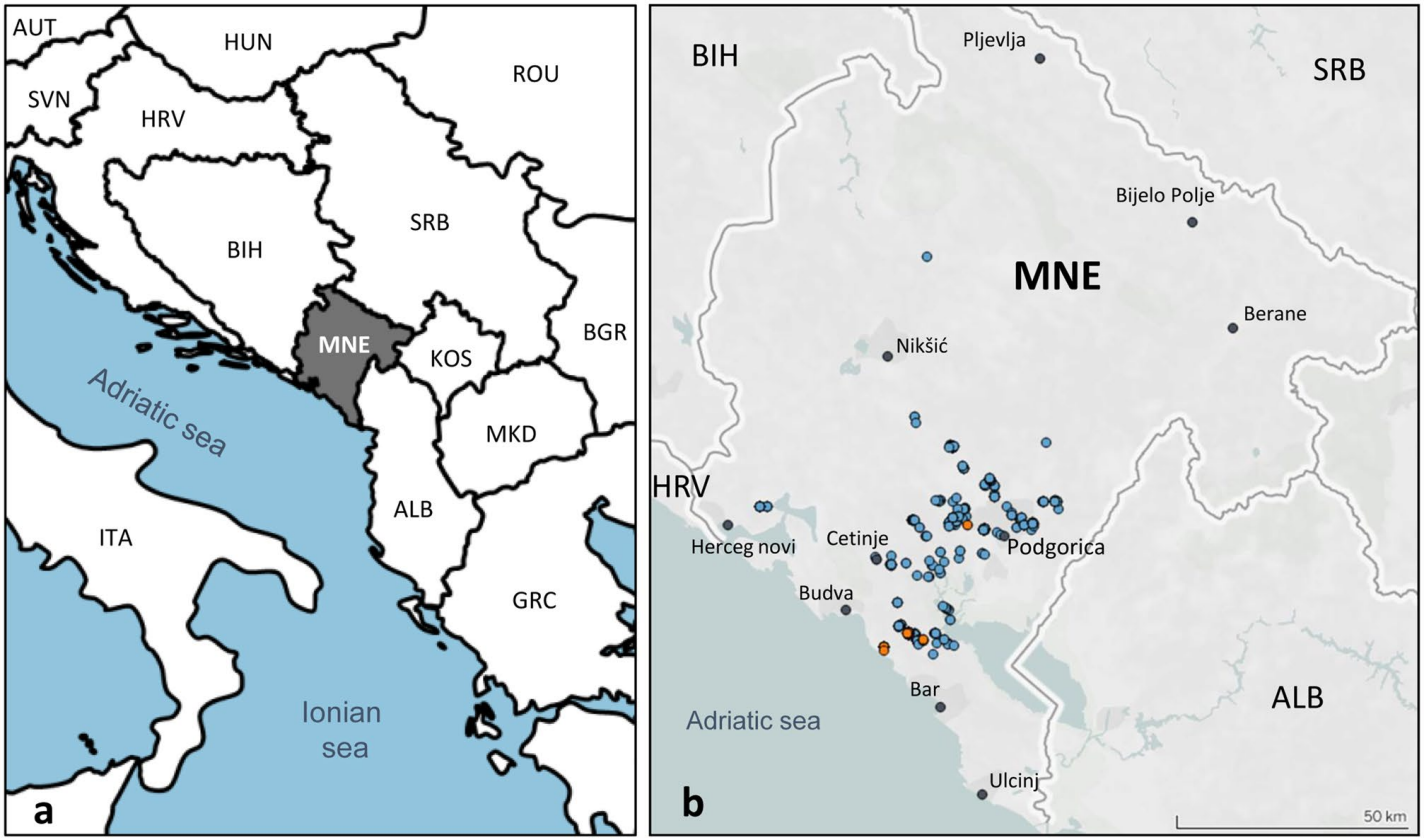
Location of Montenegro (MNE) in the Western Balkans (**a**) and sampling sites of cultivated and wild grapevines in Montenegro (**b**). In (**b**), cultivated and wild vines are shown as blue and orange circles, respectively. Main Montenegrin cities are shown as grey dots. Maps were generated using MapChart (https://mapchart.net) and Tableau v. 10.3.

A broad genetic survey was performed in traditional wine regions of Montenegro. The search and sampling of cultivated plants was exclusively focused on old vines (older than 50 years, including some extremely old plants from monasteries, with written records from 1208 and 1672), located in small vineyards or orchards mostly belonging to local wine producers for self-consumption. Vines growing in the wild were mainly collected close to river or creek banks. A total of 419 vines (45 wild vines and 374 cultivated plants) were localized, georeferenced and collected throughout Montenegro (Fig. 1 and Supplementary Table S1). In addition, we included samples of 57 grapevine accessions maintained in the ex situ* Vitis* collection of the Biotechnical Faculty of the University of Montenegro (BTF collection), which was created from surveys performed between 1956 and 1960 to preserve local cultivated grapevine genetic resources. All the sampling sites of cultivated and wild grapevines are indicated in Fig. 1b.

Genotyping of these 476 samples at 48 SNP loci revealed 144 different genetic profiles, of which 111 corresponded to vines sampled in situ across Montenegro (either cultivated or wild). Considering these 111 genotypes, 68 corresponded to cultivated plants in ancient vineyards and 43 were sampled as wild vines (Supplementary Table S1). The comparison of the SNP genetic profiles with those stored in the Instituto de Ciencias de la Vid y del Vino-SNP database (ICVV-SNP database), and their corresponding SSR genetic profiles with international databases^39,40^, allowed the identification of 33 grapevine cultivars (Supplementary Table S2 and Supplementary Table S3). We detected 45 different genotypes among the 57 accessions from the BTF collection. Only 12 of these genetic profiles were also identified among the samples obtained in situ. SNP and SSR profiles allowed the identification of 25 genotypes (8 found among the in situ collected samples), which mainly corresponded to cultivars from Greece (3), Italy (3) and the former Yugoslavia (9) (Supplementary Table S1). In total, 50 genotypes could be identified and 51 could not. Considering the set of 50 identified cultivars, 29 of them corresponded to wine cultivars (58%), six to table cultivars (12%) and 15 to cultivars with a double wine/table use, according to the *Vitis* International Variety Catalogue^40^ (VIVC, www.vivc.de), (Table 1 and Supplementary Table S1). Three out of the 51 non-identified genetic profiles were found (at least) twice only in the in situ samples. Another two were found at least twice exclusively in the BTF collection, and four were found both in the in situ sampling across the country and in the ex situ sampling in the BTF collection (Table 1). All these cases suggest they have been subjected to vegetative propagation. The remaining 42 non-identified genotypes (28 from the in situ survey and 14 from the collection) were found only once in the study.

**Table 1:** Main grapevine cultivars found across Montenegro.

Cultivar name	N	Variety number *V*IVC	Country or region of origin	Use
Kratošija^a^	106	9,703	Balkan	Wine
Vranac^a^	76	13,179	Montenegro	Wine
Lisica^a^	35	6,856	Croatia	Wine
Razaklija	27	8,945	Turkey	Table
Krstac^a^	22	24,930	Montenegro	Wine
Bioka	8	4,217	Dalmatia	Wine
Čubrica	5	12,298	Montenegro	Wine
Kadarun	5	5,900	Turkey	Wine
Bratkovina Bijela^a^	4	1,660	Croatia	Wine
Chaouch Blanc	4	10,196	Turkey	Table
Sura^a^	4	–	–	–
Vela Pergola^a^	4	22,299	Croatia	Wine
Belka^a^	3	–	–	–
Isabella	3	5,560	United States of America	Table/wine
Koenigin Der Weingaerten	3	6,350	Hungary	Table/wine
Nepoznata Bijela Brijestovo	3	–	–	–
Prokupac	3	9,734	Serbia	Wine
Vulpea	3	13,186	Austria	Wine
Coarna Alba	2	2,724	Moldova	Table/wine
Crni Krstač	2	–	–	–
Loza Svetog Vasilija Ostrozkog	2	–	–	–
Muscat Hamburg	2	8,226	United Kingdom	Table/wine
Plavina Crna^a^	2	9,557	Yugoslavia	Wine
Radovaca	2	24,598	Bosnia and Herzegovina	Wine
Afus Ali	1	122	Lebanon	Table/wine
Alphonse Lavallee	1	349	France	Raisin/table/wine
Cabernet Franc	1	1927	France	Wine
Clairette Mazel	1	2,698	France	Table/wine
Heptakilo	1	5,207	Greece	Table/wine
Lagorthi^a^	1	6,665	Greece	Wine
Merlot	1	7,657	France	Wine
Pamid	1	8,899	Bulgaria	Wine
Perlona	1	9,171	Italy	Table
Raćeška	1	25,800	Montenegro	Wine
Razaklija Brijege^a^	1	–	–	–
Rkatsiteli	1	10,116	Georgia	Table/wine
Varousset	1	12,909	France	Wine
Zadrimka^a^	1	–	–	–
Zilavka	1	13,446	Bosnia and Herzegovina	Wine
Župljanka	1	13,480	Serbia	Wine

The list includes those genotypes identified as cultivated varieties according to SNP and/or SSR profiles, as well as those unidentified genotypes found at least twice across Montenegro. When available, the region of origin of the identified variety and its main use is provided according to the *V*IVC database (www.vivc.de). ^a^Indicates those cultivars also found in the ex situ* Vitis* collection of the Biotechnical Faculty of the University of Montenegro (BTF collection).

The most commonly found genotype among the 419 grapevine samples corresponded to the variety Kratošija, which was found 106 times, followed by Vranac (76), Lisica (35), Razaklija (27), Krstač (22) and Bioka (8). A high number of the 50 identified cultivars are currently considered autochthonous varieties from the Western Balkans, such as Bratkovina Bijela, Coarna Alba, Hrvatica, Kratošija, Prokupac, Vranac or Zilavka, whereas other cultivars are considered to come from Eastern countries like Greece (Heptakilo, Karystino or Muscat a Petits Grains), Turkey (Chaouch Blanc, Kadarun or Razaklija), Lebanon (Afus Ali), Armenia (Krivalja Bijela), Azerbaijan (Sysak) and Georgia (Rkatsiteli). In addition, cultivars from Western countries like France (e.g.: Bicane, Cabernet Franc and Merlot), Germany (e.g.: Mueller Thurgau), Italy (e.g.: Malvasia Bianca Lunga) and Austria (e.g.: Silvaner Gruen) were also identified, as well as bred cultivars like Angelo Pirovano and Perlona, and *Vitis* spp. interspecific hybrid direct-producers like Isabella and Varousset.

All the wild plants hold unique genotypes, but for two genotypes (named Wild MNE 273 and Wild MNE 359) that were found twice in the wild (Supplementary Table S1). Wild MNE 359 samples were found extremely close in the municipality of Gornji Ulići, so it is probable that both samples were inadvertently taken from different branches of the same individual. Wild MNE 273 samples were found at the two sides of a road in Crmnica, suggesting the occurrence of natural vegetative multiplication^41^.

Lastly, chlorotype analyses revealed a majority of cultivars bearing chlorotypes C (43 genotypes, 42.6%) and D (33, 32.7%), followed by chlorotypes A (13, 12.9%) and B (3, 3.0%). On the contrary, most plants sampled in the wild bear chlorotype A (30 genotypes, 69.8%), followed by chlorotypes D (7, 16.7%) and C (6, 13.9%) (Supplementary Table S1).

### Population structure analysis of cultivated and wild grapevines in Montenegro

**Figure 2:**
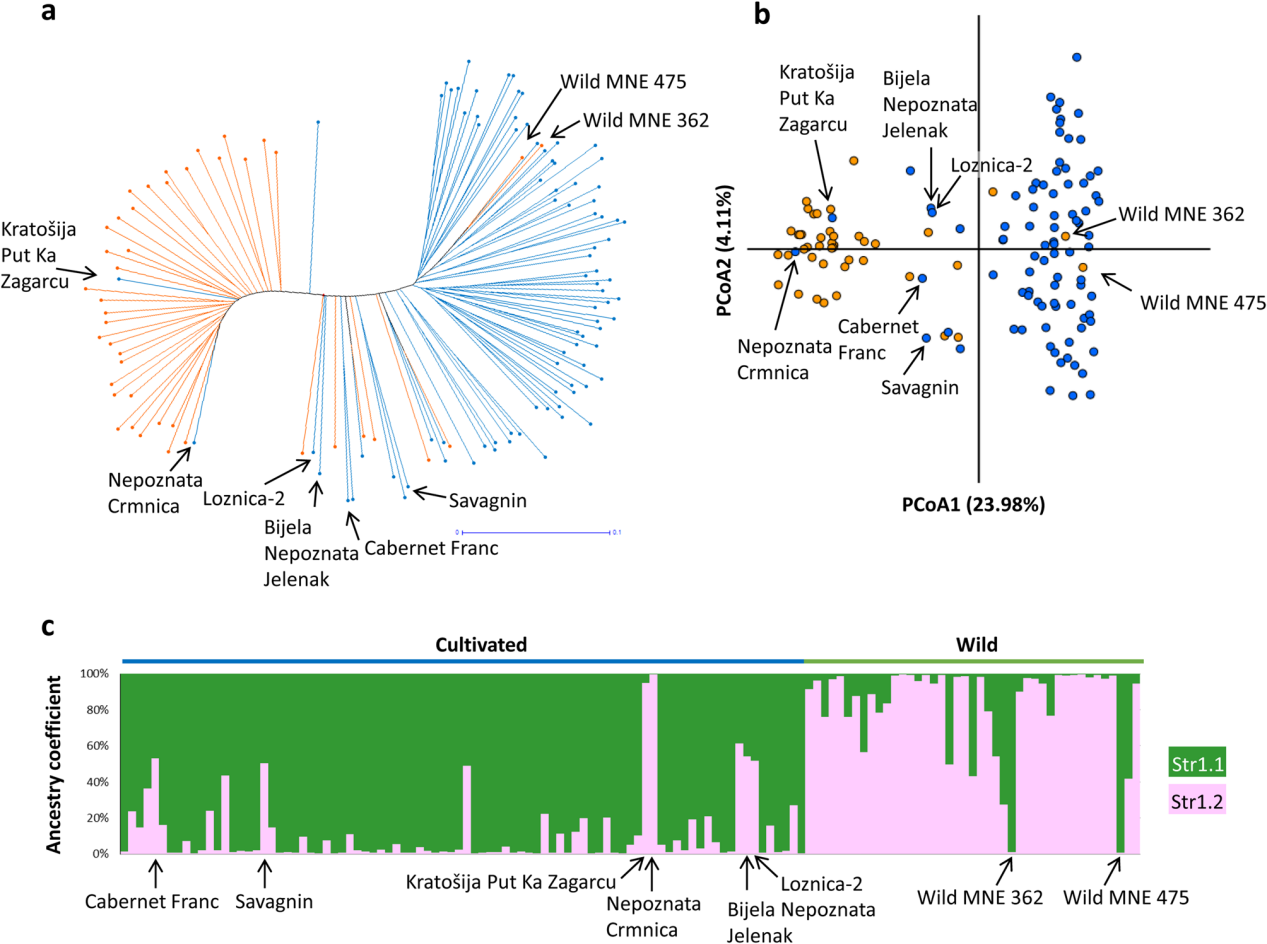
Population structure analysis of 131 non-redundant grapevine genotypes found in Montenegro. In (**a**), an unweighted neighbor-joining (UwNJ) radiation tree showing the relationship between wild vines (orange dots) and cultivated vines (blue dots) is shown. In (**b**), a principal coordinate analysis (PCoA) with both vine types in the same colors is shown. The % variance explained by the PCoA1 and PCoA2 is indicated in the plot axes. Both (**a**,**b**) plots were obtained from a dissimilarity matrix calculated in DARwin using 194 SNPs. In (**c**), STRUCTURE analysis revealing the existence of two major genetic groups, mainly corresponding to varieties sampled as cultivated and wild respectively. Every non-redundant genotype is shown as a vertical line, with color segment lengths proportional to their inferred ancestry to Str1.1 and Str1.2, (shown in green and pink, respectively). The optimal number of genetic groups (2) was set considering the Δ*K* criterion^65^. Considering a critical ancestry coefficient of *q* ≥ 0.50, 87 and 44 genotypes were assigned to Str1.1 and Str1.2, respectively.

To understand the population structure of Montenegrin grapevines, 144 non-redundant genotypes were profiled with 192 additional SNP loci. It yielded satisfactory results for 131 genotypes, which were used for further population structure, genetic diversity and pedigree analyses (Supplementary Table S2). The UwNJ distance tree generated two main clusters (Fig. 2a), which mostly reflected the sampling origin of the genotypes included in the analysis. Thus, one of the clusters included most of the genotypes collected as wild vines, whilst the other included most of the genotypes collected as cultivated plants. Interestingly, two genotypes sampled as cultivated plants (named Kratošija Put Ka Zagarcu and Nepoznata Crmnica) located in the cluster of wild vines, whereas two genotypes collected in the wild (Wild MNE 362 and Wild MNE 475) located in the cluster of cultivated vines. PCoA results supported this major genetic differentiation, with PCoA1 clearly separating the genotypes collected in the wild from those collected as cultivated vines (Fig. 2b). Clustering results for Kratošija Put Ka Zagarcu, Nepoznata Crmnica, Wild MNE 362 and Wild MNE 475 were also supported by the PCoA.

On the other hand, STRUCTURE analysis and the Δ*K* criterion suggested *K* = 2 as the optimal uppermost hierarchical level of structure for the set of 131 non-redundant grapevine genetic profiles (Supplementary Figure S1a). At this level of structure, a clear separation between the genotypes sampled as cultivated or wild was also obtained. Considering a membership coefficient (*q*-value) threshold of 0.5 for group assignment, one genetic group (Str1.1; n = 87) is mainly formed by samples collected as cultivated plants (81 genotypes, 93.1%). The other genetic group (Str1.2, n = 44) is mainly formed by genotypes sampled as wild vines (37 genotypes, 84.1%) (Fig. 2c and Supplementary Table S2). As previously noted, Kratošija Put Ka Zagarcu and Nepoznata Crmnica were found in the genetic group Str1.2 with ancestry *q* values over 0.95, whereas Wild MNE 362 and Wild MNE 475 were found in Str1.1 with ancestry *q* values over 0.98. Interestingly, two grapevine cultivars reported as progenitors of many other relevant varieties (Cabernet Franc and Savagnin), clustered in Str1.2 with most of the wild vines included in this work, with membership coefficients of 0.530 and 0.505, respectively (Supplementary Table S2).

Genetic diversity parameters were individually calculated for Str1.1 and Str1.2, excluding clearly admixed genotypes by using a more stringent threshold for group assignment (*q *values > 0.6). Significant differences (*p* ≤ 0.05) between the non-redundant genotypes assigned to Str1.1 and Str1.2 were found for all the parameters (Table 2). The group of cultivated plants (Str1.1, n = 82) showed significantly higher values of expected (*UH*_*e*_) and observed (*H*_*o*_) heterozygosity than the group of wild vines (Str1.2, n = 38), while the fixation coefficient (*F*_*IS*_) was close to zero for both genetic groups. The *F*_*ST*_ value between Str1.1 and Str1.2 populations was 0.131.

**Table 2:** Genetic diversity parameters (mean ± SD) for the two major genetic groups detected by STRUCTURE.

Population	*N*	*N*_*SNP*_	*N*_*pSNP*_	*N*_*e*_	*I*	*H*_*o*_	*UH*_*e*_	*F*_*IS*_
Str1.1 (cultivated)	82	235	235	1.58 ± 0.33	0.50 ± 0.19	0.35 ± 0.17	0.34 ± 0.15	− 0.05 ± 0.15
Str1.2 (wild)	38	231	214	1.44 ± 0.33	0.42 ± 0.20	0.27 ± 0.16	0.27 ± 0.16	− 0.01 ± 0.20
Significance	–	–	–	*	*	*	*	*

Str1.1 and Str1.2 largely divide the sampled Montenegrin genotypes as cultivated or wild, respectively. Genotypes were assigned to a genetic group if *q* ≥ 0.60. *N *sample size, *N*_*SNP*_ number of called SNPs, *N*_*pSNP*_ number of polymorphic SNPs, *N*_*e*_ number of effective alleles, *I* information index, *H*_*o*_ observed heterozygosity, *UH*_*e*_ unbiased expected heterozygosity, *F*_*IS*_ fixation coefficient. Genetic diversity parameters were calculated considering polymorphic SNPs.

*Significant differences between cultivated and wild genetic groups (*t* test; *p* ≤ 0.05).

**Figure 3:**
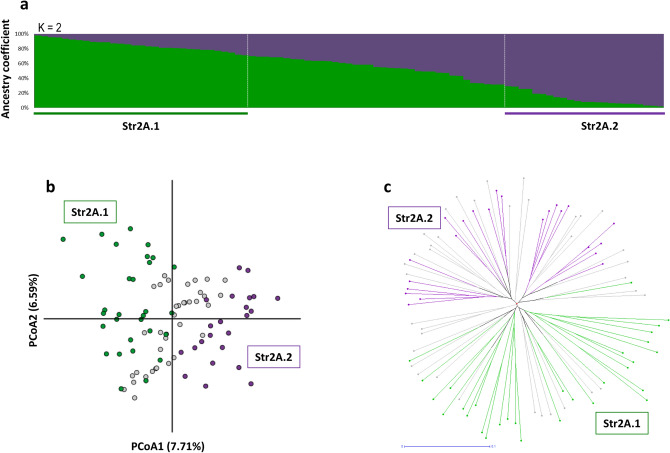
Population structure analysis of 91 non-redundant cultivated grapevine genotypes found in Montenegro. In (**a**), STRUCTURE analysis revealed the existence of two major genetic groups (Str2A.1 and Str2A.2). Every non-redundant genotype is shown as a vertical line, with color segment lengths proportional to their inferred ancestry to Str2A.1 (green) and Str2A.2 (purple). The optimal number of genetic groups (K = 2) was set considering the Δ*K* criterion^65^. Considering a critical ancestry coefficient of *q* ≥ 0.70, 31 and 23 genotypes were assigned to Str2A.1 and Str2A.2, respectively (37 genotypes were considered as admixed). In (**b**), a principal coordinate analysis (PCoA) obtained from a dissimilarity matrix calculated in DARwin from genetic data (194 SNPs) from the non-redundant genotypes is shown. The variance explained by the PCoA1 and PCoA2 is indicated as %. In (**c**), the unweighted neighbor-joining (UwNJ) radiation tree obtained for the same dataset by means of DARwin is shown. In (**b**,**c**), genotypes assigned to Str2A.1 and Str2A.2 are shown as green and purple dots, respectively (admixed genotypes are shown in grey).

Additional levels of genetic stratification in the set of cultivated vines were tested through a second STRUCTURE analysis, considering the 87 non-redundant genetic profiles assigned to Str1.1 (*q *value > 0.5) and the four genotypes sampled as cultivated but assigned to Str1.2 with *q* value < 0.6 (Savagnin, Loznica-2, Cabernet Franc and Bijela Nepoznata Jelenak, Supplementary Table S2). The Δ*K* criterion reported similar values at *K* = 2 and *K* = 3 (Supplementary Figure S1b). At *K* = 2, considering a critical *q* value of 0.70 for group assignment, the two genetic groups included 54 genotypes (Fig. 3a and Supplementary Table S2). Str2A.1 contained 31 cultivars of diverse origin, but most of them ancient and progenitors of many other relevant grapevine varieties (e.g.: Cabernet Franc, Heptakilo, Muscat a Petits Grains, Savagnin). Str2A.2 (n = 23) mostly included cultivars considered autochthonous from the Western Balkans (e.g.: Coarna Alba, Čubrica, Kratošija, Raćeška, Vranac). This level of structuring was supported by the UwNJ and PCoA analyses (Fig. 3b,c), with genotypes assigned to Str2A.1 or Str2A.2 separated by PCoA1 and PCoA2 (Fig. 3b), and clustering in opposite branches of the UwNJ distance tree (Fig. 3c). At *K* = 3, three genetic groups clustering 30 genotypes were obtained (*q *value > 0.70 for group assignment) (Supplementary Figure S2a and Supplementary Table S2). Str2B.1 contained 7 genotypes (“RAZ-group”), related to the ancient Turkish cultivar Razaklija (prime name in *V*IVC: Parmak Cerven^40^). Str2B.2 included 6 genotypes native to the Western Balkans (“KRA-group”), such as Kratošija and Vranac. Interestingly, these 6 genotypes were assigned to Str2A.2 when considering *K* = 2. Str2B.3 is formed by 17 genotypes, including internationally renowned cultivars like Cabernet Franc, Merlot, Mueller Thurgau, Savagnin or Silvaner Gruen (“INT-group”), and all were assigned to Str2A.1 when considering *K* = 2. PCoA results supported the genetic differentiation indicated by STRUCTURE at *K* = 3, with genotypes assigned to RAZ-group, KRA-group and INT-group clustering in different regions of the plot (Supplementary Figure S2b). Nevertheless, we did not obtain a clear separation of these 30 genotypes in the UwNJ distance tree, as genotypes assigned to the RAZ-group were scattered in the tree (Supplementary Figure S2c).

### Parentage analysis of cultivated and wild grapevines in Montenegro

**Figure 4:**
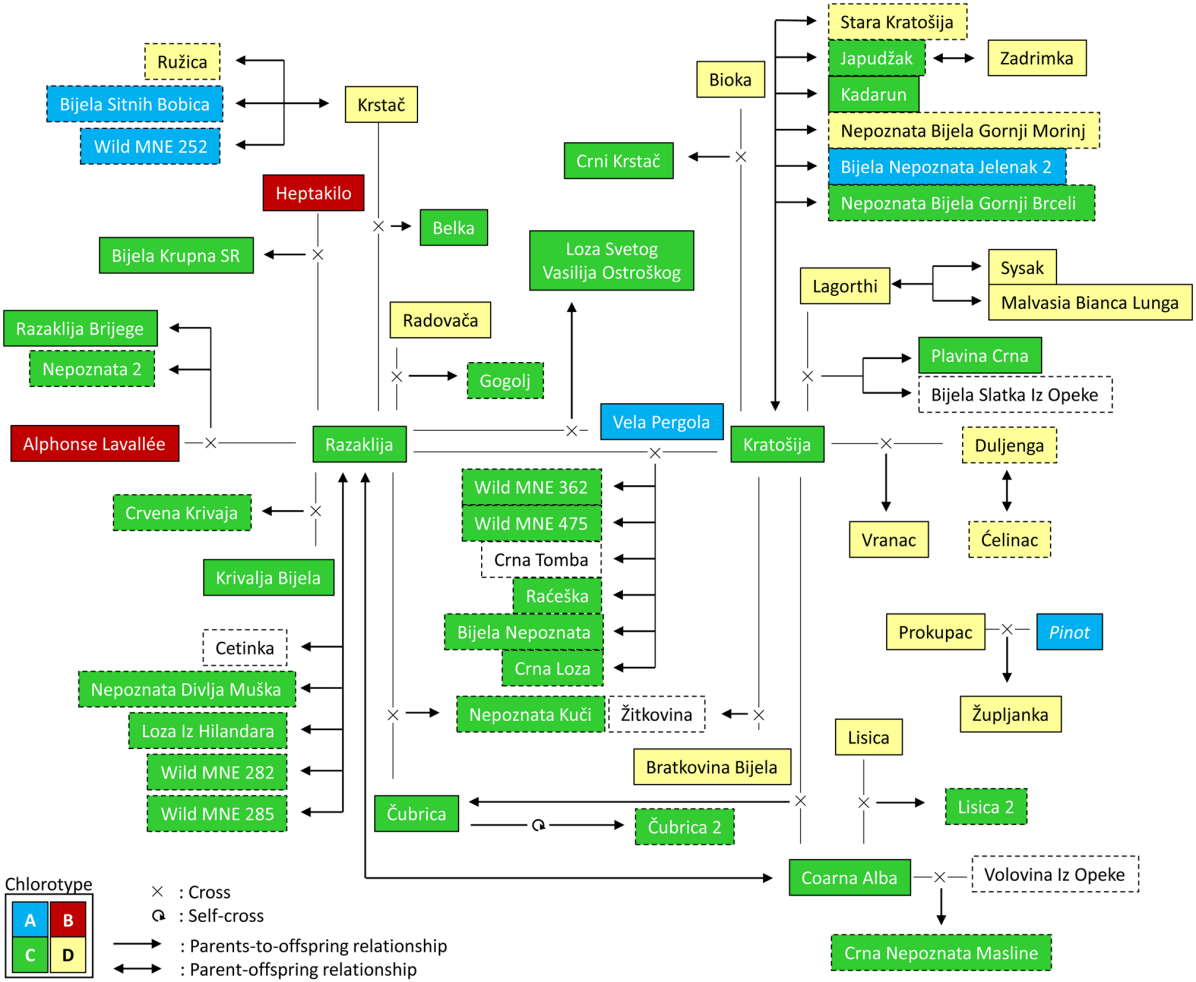
First-order genetic relationships (trios and duos) detected for cultivated and wild grape varieties sampled in Montenegro. Chlorotypes (A, B, C or D) are indicated with different colors, according to the inserted code. If white, no information on chlorotype was available. Unidentified and unique genotypes in the ICVV-SNP database are shown in boxes with broken borders. These genetic relationships were obtained with the likelihood-based method implemented in Cervus v.3.0 for parentage analysis, on the basis of SNP genetic data.

The 131 non-redundant grapevine genetic profiles were merged with those stored in the ICVV-SNP database for a wide search of possible first-order kinship relationships using 240 SNP-profiles. All reliable trios (mother + father + offspring) and duos (parent-offspring) involving (at least) one of the genotypes found in this work can be found in Supplementary Tables S4A and S4B, together with their LOD values and the number of mismatching loci. The most relevant relationships resulting from our analysis are shown in Fig. 4. Global results showed 25 compatible trios, with high LOD values (> 62.50) and a maximum of 2 mismatching loci. Our results confirm the pedigrees previously suggested for Clairette Mazel^42^, Plavina Crna^42^ and Župljanka^43^. Results indicate a leading role of Razaklija and Kratošija in the generation of Montenegrin grapevine diversity, being involved as progenitors in 14 and 12 pedigrees, respectively. Together they have six offspring, including two genotypes sampled as wild vines (Wild MNE 362 and Wild MNE 475, with ancestry *q*-values over 0.98 in Str1.1). In addition, we detected six compatible parent–offspring relationships for Kratošija, and five for Razaklija, including other two genotypes sampled as wild vines (Wild MNE 282 and Wild MNE 285, with ancestry *q* values *ca.* 0.50 in Str1.1). Pedigree analysis also allowed discovering the genetic origin of Vranac, currently the most emblematic variety in Montenegro. Our results confidently support that Vranac derived from a hybridization event between cultivars Duljenga and Kratošija (LOD = 85.10), with no mismatching loci in the proposed trio. This pedigree was also supported by an additional set of 20 SSR markers (Supplementary Table S5), and chlorotype analyses identified Duljenga as the female progenitor of Vranac (Supplementary Table S4A). In addition, we identified one cultivated genotype (Čubrica-2) as the compatible result of a self-cross of cultivar Čubrica (Fig. 3). No full trios were found among the wild vines, but two reliable duos were detected in two different populations: in Lesendro Fortress (involving Wild MNE 275 and Wild MNE 276) and in Orasi (involving Wild MNE 363 and Wild MNE 364) (Supplementary Table S4B).

## Discussion

Modern Montenegrin viticulture and winemaking industries co-exist with a significant number of old and small vineyards managed through traditional techniques^14,15^. Such viticultural tradition was shaped by a unique geographic position, terrain fragmentation and history. This traditional activity provides a fascinating window into this relict viticulture, and represents a unique opportunity to explore how viticulture evolved before wine market standardization. To this aim, we have studied the diversity of the traditional Montenegrin grapevine genetic pool through the most extensive search and sampling of cultivated and wild grapevines ever done in the country so far.

We found an unexpectedly high genetic variability in the cultivated set, detecting 101 genotypes. Fifty genetic profiles were identified as well-known wine, table or multiple-use grape cultivars, most of which were Western Balkan autochthonous varieties. Kratošija and Vranac, the most important cultivars for current Montenegrin viticulture, were also the two most commonly found genotypes across old Montenegrin vineyards, reflecting their relevance in the past century^10,17^. In addition, we identified a significant number of foreign cultivars, supporting historical reports that indicate multiple introduction events in the country from diverse regions at different times and with different aims^6^. Cultivars from Eastern regions are possibly a consequence of viticulture dissemination from middle-East regions to Western European countries^2^, whilst cultivars from Western regions mostly derive from the intense exchange of plant material happened during the second half of the nineteenth century to fight grape phylloxera, mildews, and other pests^2^.

Furthermore, we found 51 unidentified genotypes, cultivated in many cases as homonyms, under wrong cultivar names. Nine of them were found in at least two different sampling points (including the BTF collection), indicating their vegetative propagation and, therefore, that they are true cultivars, on the brink of extinction. Interestingly, some of them were found to be offspring of other well-known local cultivars (e.g.: Belka, Crni Krstač), indicating a Montenegrin origin. The remaining 42 non-identified genotypes only appeared once in the sampling of cultivated plants. Some of them may represent true varieties too, as supported by the genetical identification of descendants for some of these genotypes (e.g.: Crna Nepoznata Malisne for Volovina Iz Opeke). As they were sampled only once, these 42 genotypes are examples of critically endangered Montenegrin grapevines. Besides, a remarkable particularity of Montenegro viticulture is the current presence of an important number of putative ‘proto-varieties’. This term is used here to designate plants that have been only cultivated by local grape growers, i.e. plants that directly grow from seeds, or have been multiplied through cuttings just once, from the place where the seed germinated to the orchard. Eventually, they could be multiplied and distributed, becoming varieties. This is how most of the varieties originated in the past, but this process is not active anymore in Western European regions.

Strikingly, an important number (33) of the genotypes conserved in the BTF collection (from a sampling of the 1960’s) was not found among the in situ samples studied in this work, indicating fast disappearance of ancient varieties, and underscoring the relevance of these surveys to preserve local genetic resources from extinction^19^.

Sexual reproduction is known to be the prime process in the generation of new grapevine genotypes^2^, and autochthonous varieties are the main source for shaping local grapevine diversity. Previous works have identified intricate parentage networks linking multiple grapevine cultivars^1, 42^, and major founders have been detected in many traditional winemaking regions, such as Pinot and Gouais Blanc (synonym Heunisch Weiss) in Burgundy and in Northeastern France^44^, Cabernet Franc in the Aquitania region of Bordeaux^45^, Hebén in the Iberian Peninsula^46,47^, and Sangiovese and Garganega in the Italic Peninsula^48^. Here, we found that Razaklija and Kratošija played a key role shaping the grapevine genetic pool of Montenegro, participating in a high number of the discovered trios and duos. Razaklija is a Turkish table grape cultivar existing in many grapevine collections under multiple synonyms^40^, and is a well-known genitor of several Albanian cultivars^12^. We found it compatible as the female parent in 14 full pedigrees (as expected, given its functional female flowers^40^). Interestingly, several grapevine varieties with female flowers have been previously reported to have an important contribution in the establishment of local genetic networks, like Hebén and Marufo in the Iberian Peninsula^38,47^. Unlike hermaphrodite plants, females need to cross-pollinate to produce descendants, process that increases genetic diversity and increases hybrid plant vigor^38^, which could have favored their selection as seed donors by early farmers to ensure grape production.

Kratošija (also known as Zinfandel in California, Primitivo in Italy and Tribidrag, Pribidrag or Crljenak Kastelanski in Croatia^32^) is a cultivar grown in the Western Balkans for centuries. Nevertheless, its place of origin is controversial, and it has changed as new evidence was added: first from USA to Italy^49^, and then to Croatia, where old references and a reduced number of plants with a matching genotype were found under the name Crljenak Kastelanski^50^. More recently, the first molecular analysis of Kratošija proved that it has the same genotype^32^. In our study we identified 106 plants of Kratošija in old Montenegrin vineyards, and we found it to be the genitor (trios and duos) of almost 20 grapevine genotypes cultivated in Montenegro. In accordance with its ancient cultivation in this region, multiple Kratošija biotypes differing in traits like grape yield, cluster compactness or cluster size have been found in Montenegro^14^. In addition, early historical references already indicate the widespread use of Kratošija grapes for traditional winemaking in Montenegro, as reported in the Medieval Statute of Budva^9^, and reflected in folklore traced back to the eighteenth century^10^. Although definitive proof to establish the birthplace of an ancient variety cannot be provided, our results and the mentioned evidence support that Kratošija might have originated somewhere within Montenegro, agreeing with previous findings^51^.

Vranac is considered an old autochthonous Montenegrin grape variety used for local red wine production for centuries^10^, and it was already described as a black-berried Montenegrin cultivar in the *Traité général de viticulture* of Viala and Vermorel (1909) under the name “Vranatz-Krstatch”^52^. Previous reports indicate the existence of a first-degree relationship between Kratošija and Vranac^32^. Here, we found the full pedigree for Vranac, being the result of a compatible offspring between Duljenga and Kratošija. Duljenga was exclusively found in the BTF collection, and there are no reliable documents indicating the origin of this cultivar. Its presence in the BTF collection and its role as Vranac progenitor indicates that it was cultivated at some time in Montenegro.

As nicely summarized by Miller and Gross, the domestication process in perennial fruit crops produces a *continuum* of plant populations, which range from exploited wild individuals to initial domesticates to cultivated populations^53^. In agreement with surveys done in wider frameworks^20,34,54^, the clear division between Str1.1 and Str1.2 seems to correspond to the two *V. vinifera* L. subspecies: *sativa* and *sylvestris*. However, it does not completely correspond with cultivated and wild conditions, as some intermediate genotypes generating a *continuum* were found. Population analyses allowed us to identify two exploited *sylvestris* plants (Kratošija Put Ka Zagarcu and Nepoznata Crmnica) collected from vineyards and yet genetically of the subsp. *sylvestris*. These plants bear chlorotype A and unique genotypes with no matches in the consulted databases, indicating they arise from *sylvestris* vines that were vegetatively propagated. These exploited *sylvestris* plants could be also considered as proto-varieties and, unlike the cases mentioned above, may represent novel active domestication events. Previous studies also report the in situ domestication of *sylvestris* autochthonous vines for grape production^38,55^, suggesting the existence of secondary domestication events in areas of grapevine cultivation at different times. The existence of these events is an open issue in grapevine research^56^, being difficult to ascertain if current cultivated grapevines are the result of multiple domestication events^37,54^ or a combination of a primary domestication event and multiple local introgression events during the geographic expansion of the crop to new locations.

We found a set of cultivated plants with similar percentages of ancestry to the *sativa* and *sylvestris* genetic subgroups (like Bijela Nepoznata Jelenak and Loznica-2). As stated by Stebbins in 1950, any single valuable hybrid individual, once obtained, can immediately become the progenitor of a new variety and can be perpetuated indefinitely^53^. In addition to Bijela Nepoznata Jelenak and Loznica-2, this fact was also observed with two widespread cultivars, Cabernet Franc and Savagnin (synonym Traminer). These two cultivars are genitors of a high number of cultivars (20 for Cabernet Franc and 70 for Savagnin^40^), and recent paleogenomic analyses indicate that Savagnin could have been uninterruptedly cultivated in Europe for (at least) 900 years^4^. The number of sexual cycles separating domesticated individuals from their wild ancestors is suggested to be low in perennial plants with clonally-propagated varieties^53^, which might explain the high level of wild ancestry detected in these two ancient varieties. In that sense, Bijela Nepoznata Jelenak and Loznica-2 are examples of active pathways to introgress genomic regions of subsp. *sylvestris* into new varieties.

Finally, we detected six individuals sampled as wild vines with higher *sativa* than *sylvestris* genetic composition (four of them bearing chlorotype C). Two of them (Wild MNE 362 and Wild MNE 475) show extremely high coefficients of ancestry in the genetic subgroup of cultivated varieties and are compatible offspring of Kratošija and Razaklija, which classifies them in the *sativa* subspecies. Possibly, they have derived from seeds produced in vineyards and dispersed into neighboring natural environments by humans or animals. The presence of naturalized vines from grapevine cultivars, rootstocks and/or direct-producer hybrids is well documented in alluvial European forests^57,58^, and they are currently considered as invasive plants capable of contaminating natural wild grapevine populations. Thus, the cultivated vines (human-cared vines) do not always belong to the *sativa* subspecies, just as vines growing wild without cares do not always belong to the *sylvestris* subspecies.

Our in-depth study of *V. vinifera* L. diversity in traditional viticultural regions across Montenegro provided insights not only about the existing genetic diversity, but also on how it was generated. The exploration of these unique regions, shaped by specific geographic and historical conditions, made it possible to detect different stages in the domestication process of this woody perennial, providing useful information on the processes of evolution underlying the generation of the current cultivated varieties. The study allowed us to conclude that the high diversity observed for Montenegrin varieties arose from ancient pathways to generate genetic diversity that are still active. These pathways involved the cultivation of new grape plants from seeds which increased genetic variation by: (1) the spontaneous sexual hybridization among cultivars introduced from different regions, (2) the spontaneous sexual hybridization between *sylvestris* and *sativa* grapevines, and (3) the vegetative reproduction of local *sylvestris* plants for grape production.

## Materials and methods

### Collection of plant material

A total of 419 cultivated (374) and wild (45) grapevines were sampled throughout Montenegro, focusing in old, traditional vineyards dispersed in the two major viticulture regions of the country (the Lake Skadar basin and the coastal region). Samples were collected in several exploration trips in 2013, 2014, 2016 and 2017, and GPS coordinates were recorded for each sampling site (Fig. 1 and Supplementary Table S1). Young leaves were collected on site for each sample, and kept in ice until storage at − 80 °C for DNA extraction and genotyping. In addition, a set of 57 samples from the ex situ* Vitis* collection of the Biotechnical Faculty of the University of Montenegro (BTF collection) was included as a reference to aid in the identification of autochthonous cultivars. Cuttings from cultivated samples were taken in winter, grafted and planted in a new repository created by 13 Jul Plantaže to preserve this material. Cuttings from wild samples will be collected in upcoming seasons, and treated as described to be part of this repository.

### DNA isolation, genotyping and varietal identification

DNA was isolated from frozen leaves as previously detailed^59^. All samples were initially profiled for a core set of 48 nuclear SNP markers^29^, using the SNP-genotyping services provided by the Spanish Centro Nacional de Genotipado (CEGEN), as recently described^38^ or the Sequencing and Genotyping Unit of the University of the Basque Country, using Fluidigm technology. Three chloroplast SNPs that allow to distinguish the main grapevine chloroplast haplotypes (A, B, C and D)^36^ were used to determine sample chlorotype. Non-redundant genetic profiles (144) for the 48 SNPs were pair-wise compared with the ICVV-SNP database for cultivar identification, which includes the genetic profiles of more than 2,800 non-redundant genotypes for 48 SNPs. In those cases in which the SNP profile did not match with an identified variety in the ICVV-SNP database, samples were additionally characterized with 9 SSR markers (*VVS2*, *VVMD5*, *VVMD7*, *VVMD25*, *VVMD27*, *VVMD28*, *VVMD32*, *VrZAG62* and *VrZAG79*) in the genotyping platform of the Centro de Investigación Biomédica de La Rioja (CIBIR). Profiles of SSRs were compared with those stored in the *V*IVC^40^ and the European *Vitis* Database^39^. All 144 non-redundant genotypes were profiled for an additional set of 192 SNP markers^60^ using the genotyping platforms stated before. Unfortunately, no satisfactory results were obtained for 13 genotypes, so 131 non-redundant grapevine genotypes with genetic profiles for 240 SNP loci were used for population structure and genetic diversity analyses. SNP and SSR analyses were conducted as previously described^38, 61,62^.

### Analysis of population structure and genetic diversity parameters

An Unweighted Neighbour-Joining (UwNJ) distance tree and a Principal Coordinate Analysis (PCoA) were calculated to explore the relationship between the cultivated and wild grapevine genotypes collected across Montenegro. To this aim, a dissimilarity matrix with 10.000 bootstrap steps was calculated using the DARwin software package v. 6.0.21^63^ for 131 non-redundant genotypes considering 194 SNPs (SNPs with missing data in 10 or more grapevine genotypes were discarded). This dissimilarity matrix was used for PCoA and UwNJ analyses; the UwNJ tree was constructed on the basis of 1.000 bootstrap replicates.

Then, the Bayesian clustering method implemented in the STRUCTURE v.2.3.4 software^64^ was used to infer the number of genetic groups present in the set of 131 non-redundant grapevine genotypes. Here, we tested the existence of genetic structure considering a number of hypothetical genetic groups (*K*) ranging from 1 to 10, using a cycle of 100.000 burn-in steps followed by 150.000 Markov Chain Monte Carlo repetitions. To assess the consistency of the results, 10 runs per *K* value were performed, each one considering an admixture model with correlated allele frequencies among populations. The most likely number of genetic groups was set using the Δ*K* criteria^65^ implemented in STRUCTURE HARVESTER v. 0.6.94^66^, and CLUMPP v.1.1.2^67^ was used to align the 10 different runs. Genotypes were assigned to a genetic group considering a threshold of *q* ≥ 0.50. Results were graphically represented by means of the web-based software STRUCTURE PLOT v.2.0^68^.

Genetic diversity parameters (number of effective alleles (*N*_*e*_), information index (I), observed heterozygosity (*H*_*o*_), unbiased expected heterozygosity (*UH*_*e*_) and fixation coefficient (*F*)) were calculated for the genotypes assigned to the two genetic groups identified by STRUCTURE, discarding those identified as *Vitis* spp. interspecific hybrids for their non-*vinifera* genetic background. To avoid the effect of individuals with a similar *q* ancestry value in both genetic groups, we set a *q* value threshold of 0.60 for group assignation. Thus, cultivated and wild genetic groups included 82 and 38 non-redundant genotypes, respectively. The *F*_*ST*_ statistic was used to analyze the genetic distance between cultivated and wild genotypes. Non-polymorphic markers (5 and 26 for the cultivated and wild subsets, respectively) were excluded for diversity parameters estimation. Calculations were performed using GenAlEx v. 6.5^69^. As previously indicated^61^, mean values of these metrics were subjected to a *t *test analysis to detect significant differences between both groups, considered significant at *p* < 0.05. This analysis was performed using IBM SPSS Statistics v.25.0 (Chicago, IL, USA).

Lastly, we performed a second round of hierarchical STRUCTURE analysis to evaluate additional levels of genetic stratification^70^, focusing on the inferred genetic group of cultivated grapevines (91 non-redundant genotypes). The same procedure described above was used, but we selected a more stringent *q* value (0.70) as group assignation threshold. The consistency of the genetic structure results obtained in this second round was evaluated through a PCoA and an UwNJ analysis, performed as detailed before.

### Parentage analysis

Non-redundant grapevine genotypes were merged with those of the ICVV-SNP database to complete 1921 genotypes with data for 240 SNPs and analyzed to detect possible first-order kinship relationships (trios and parent–offspring pairs), using the likelihood-based method implemented in Cervus v. 3.0^71^ as previously detailed^38,61^. The likelihood of each detected trio and parent–offspring pair (duo) was evaluated considering the natural logarithm of the overall likelihood ratio (LOD) score, and a maximum number of mismatching loci of 1 or 2 SNPs for duos and trios, respectively. For each trio, chlorotypes were used to determine which of the putative parents acted as mother, according to the maternal transmission of chloroplasts in grapevine^36^.

## Supplementary information

Supplementary Figures

Supplementary Tables

## References

[1] Myles S (2011). Genetic structure and domestication history of the grape. Proc. Nat. Acad. Sci. USA.

[2] This P, Lacombe T, Thomas MR (2006). Historical origins and genetic diversity of wine grapes. Trends Genet..

[3] Cvijić J (1918). The zones of civilization of the Balkan Peninsula. Geogr. Rev..

[4] Ramos-Madrigal J (2019). Palaeogenomic insights into the origins of French grapevine diversity. Nat. Plants.

[5] Garnier N, Valamoti SM (2016). Prehistoric wine-making at Dikili Tash (Northern Greece): integrating residue analysis and archaeobotany. J. Archaeol. Sci..

[6] Štajner N (2014). Microsatellite inferred genetic diversity and structure of Western Balkan grapevines (*Vitis vinifera* L.). Tree Genet. Genomes.

[7] Pilipovic S (2013). Wine and the vine in Upper Moesia. Archeological and epigraphic evidence. Balcanica.

[8] Marković, Č. *Antićka Budva Nekropole Istraživanja 1980–1981* (Matica Crnogorska, Podgorica, 2012).

[9] Ljubić, S. *Statuta et leges civitatis Buduae, civitatis Scardonae, et civitatis et insulae Lesinae. Opera prof. Simeonis Ljubić*. (Officina Societatis Typographicae, Zagreb, 1882-3).

[10] Maraš V (2015). Origin and characterization of Montenegrin grapevine varieties. Vitis.

[11] Tello J, Mammerler R, Cajic M, Forneck A (2019). Major outbreaks in the nineteenth century shaped grape phylloxera contemporary genetic structure in Europe. Sci. Rep..

[12] Carka F, Maul E, Sevo R (2015). Study and parentage analysis of old Albanian grapevine cultivars by ampelography and microsatellite markers. Vitis.

[13] Štajner N, Angelova E, Bozinovic Z, Petkov M, Javornik B (2009). Microsatellite marker analysis of Macedonian grapevines (*Vitis vinifera *L.) compared to Bulgarian and Greek cultivars. J. Int. Sci. Vigne Vin..

[14] Maraš V, Bozovic V, Giannetto S, Crespan M (2014). SSR molecular marker analysis of the grapevine germplasm of Montenegro. J. Int. Sci. Vigne Vin..

[15] Maraš V, Morata A (2019). Ampelographic and genetic characterization of Montenegrin grapevine varieties. Advances in Grape and Wine Biotechnology, Ch. 4.

[16] FAO. *FAOSTAT*. https://www.fao.org/faostat/en/#data/QC (2019).

[17] Pajović-Šćepanović R, Wendelin S, Eder R (2018). Phenolic composition and varietal discrimination of Montenegrin red wines (*Vitis vinifera* var. Vranac, Kratošija, and Cabernet Sauvignon). Eur. Food Res. Technol..

[18] Zulj-Mihaljevic M (2015). Cultivar identity, intravarietal variation, and health status of native grapevine varieties in Croatia and Montenegro. Am. J. Enol. Vitic..

[19] Wolkovich EM, García de Cortázar-Atauri I, Morales-Castilla I, Nicholas KA, Lacombe T (2018). From Pinot to Xinomavro in the world's future wine-growing regions. Nat. Clim. Change.

[20] Drori E (2017). Collection and characterization of grapevine genetic resources (*Vitis vinifera*) in the Holy Land, towards the renewal of ancient winemaking practices. Sci. Rep..

[21] Beslic Z (2012). Genetic characterization and relationships of traditional grape cultivars from Serbia. Vitis.

[22] Sladonja B, Poljuha D, Plavsa T, Persuric D, Crespan M (2007). Autochthonous Croatian grapevine cultivar ‘Jarbola’—molecular, morphological and oenological characterization. Vitis.

[23] Štajner N (2015). Genetic clustering and parentage analysis of Western Balkan grapevines (*Vitis vinifera *L.). Vitis.

[24] Boccacci P, Torello-Marinoni D, Gambino G, Botta R, Schneider A (2005). Genetic characterization of endangered grape cultivars of Reggio Emilia province. Am. J. Enol. Vitic..

[25] Vouillamoz JF (2007). Genetic characterization and relationships of traditional grape cultivars from Transcaucasia and Anatolia. Plant Gen. Resour..

[26] De Lorenzis G (2019). SNP genotyping elucidates the genetic diversity of *Magna Graecia* grapevine germplasm and its historical origin and dissemination. BMC Plant Biol..

[27] Sefc KM, Regner F, Turetschek E, Glössl J, Steinkellner H (1999). Identification of microsatellite sequences in *Vitis riparia* and their applicability for genotyping of different *Vitis* species. Genome.

[28] This P (2004). Development of a standard set of microsatellite reference alleles for identification of grape cultivars. Theor. Appl. Genet..

[29] Cabezas JA (2011). A 48 SNP set for grapevine cultivar identification. BMC Plant Biol..

[30] Vélez MD, Ibáñez J (2012). Assessment of the uniformity and stability of grapevine cultivars using a set of microsatellite markers. Euphytica.

[31] Ibáñez J, Vélez M, de Andrés MT, Borrego J (2009). Molecular markers for establishing distinctness in vegetatively propagated crops: a case study in grapevine. Theor. Appl. Genet..

[32] Calò A, Costacurta A, Maraš V, Meneghetti S, Crespan M (2008). Molecular correlation of Zinfandel (Primitivo) with Austrian, Croatian, and Hungarian cultivars and Kratošija, an additional synonym. Am. J. Enol. Vitic..

[33] Cipriani G (2010). The SSR-based molecular profile of 1005 grapevine (*Vitis vinifera* L.) accessions uncovers new synonymy and parentages, and reveals a large admixture amongst varieties of different geographic origin. Theor. Appl. Genet..

[34] Emanuelli F (2013). Genetic diversity and population structure assessed by SSR and SNP markers in a large germplasm collection of grape. BMC Plant Biol..

[35] Bacilieri R (2013). Genetic structure in cultivated grapevine is linked to geography and human selection. BMC Plant Biol..

[36] Arroyo-García R (2002). Chloroplast microsatellite polymorphisms in *Vitis* species. Genome.

[37] Arroyo-García R (2006). Multiple origins of cultivated grapevine (*Vitis vinifera* L. ssp *sativa*) based on chloroplast DNA polymorphisms. Mol. Ecol..

[38] Cunha J (2020). Genetic relationships among Portugueses cultivated and wild *Vitis vinifera* L. germplasm. Front Plant Sci..

[39] Maul E (2012). The European *Vitis* Database (www.eu-vitis.de): a technical innovation through an online uploading and interactive modification system. Vitis.

[40] Maul, E. & Töpfer R. Vitis international variety catalogue: www.vivc.de. Accessed February 2020 (2020).

[41] D'Onofrio C (2020). Introgression among cultivated and wild grapevine in Tuscany. Front Plant Sci.

[42] Lacombe T (2013). Large-scale parentage analysis in an extended set of grapevine cultivars (*Vitis vinifera* L.). Theor. Appl. Genet..

[43] Tomic L, Stajner N, Jovanovic-Cvetkovic T, Cvetkovic M, Javornik B (2012). Identity and genetic relatedness of Bosnia and Herzegovina grapevine germplasm. Sci. Hort..

[44] Bowers JE (1999). Historical genetics: the parentage of Chardonnay, Gamay, and other wine grapes of northeastern France. Science.

[45] Bowers JE, Meredith CP (1997). The parentage of a classic wine grape Cabernet Sauvignon. Nat. Genet..

[46] Cunha J (2015). Grapevine cultivar “Alfrocheiro” or “Bruñal” plays a primary role in the relationship among Iberian grapevines. Vitis.

[47] Zinelabidine LH (2015). Pedigree analysis of the Spanish grapevine cultivar ‘Heben’. Vitis.

[48] Crespan M (2008). ‘Sangiovese’ and ‘Garganega’ are two key varieties of the Italian grapevine assortment evolution. Vitis.

[49] Bowers JE, Bandman EB, Meredith CP (1993). DNA fingerprint characterization of some wine grape cultivars. Am. J. Enol. Vitic..

[50] Maletic E (2004). Zinfandel, Dobricic, and Plavac mali: the genetic relationship among three cultivars of the Dalmatian Coast of Croatia. Am. J. Enol. Vitic..

[51] Scienza A, Imazio S (2018). La stirpe del vino.

[52] Viala, P. & Vermorel, V. Tome VII. In: *Traité général de viticulture: Ampelographie* (ed Masson et Cie) (Librairies de L'Acadêmie de Médecine, 1909).

[53] Miller AJ, Gross BL (2011). From forest to field: perennial fruit crop domestication. Am. J. Bot..

[54] Riaz S (2018). Genetic diversity analysis of cultivated and wild grapevine (*Vitis vinifera* L.) accessions around the Mediterranean basin and Central Asia. BMC Plant Biol..

[55] Grassi F (2003). Evidence of a secondary grapevine domestication centre detected by SSR analysis. Theor. Appl. Genet..

[56] Zhou Y, Muyle A, Gaut BS, Cantu D, Walker MA (2019). Evolutionary genomics and the domestication of grapes. The Grape Genome.

[57] Meléndez E (2016). Evolution of wild and feral vines from the Ega river gallery forest (Basque Country and Navarra, Spain) from 1995 to 2015. J. Int. Sci. Vigne Vin..

[58] Arrigo N, Arnold C (2007). Naturalised *Vitis* rootstocks in Europe and consequences to native wild grapevine. PLoS ONE.

[59] Tello J, Torres-Pérez R, Grimplet J, Ibáñez J (2016). Association analysis of grapevine bunch traits using a comprehensive approach. Theor. Appl. Genet..

[60] Lijavetzky D, Cabezas JA, Ibáñez A, Rodriguez V, Martínez-Zapater JM (2007). High throughput SNP discovery and genotyping in grapevine (*Vitis vinifera* L.) by combining a re-sequencing approach and SNPlex technology. BMC Genom..

[61] Ghaffari S (2014). Genetic diversity and parentage of Tunisian wild and cultivated grapevines (*Vitis vinifera* L.) as revealed by single nucleotide polymorphism (SNP) markers. Tree Genet. Genomes.

[62] Ibáñez J (2012). Genetic origin of the grapevine cultivar Tempranillo. Am. J. Enol. Vitic..

[63] Perrier, X. & Jacquemond-Collet, J. P. DARwin software. https://darwin.cirad.fr (2006).

[64] Pritchard JK, Stephens M, Donnely P (2000). Inference of population structure using multilocus genotype data. Genetics.

[65] Evanno G, Regnaut S, Goudet J (2005). Detecting the number of clusters of individuals using the software STRUCTURE: a simulation study. Mol. Ecol..

[66] Earl D, vonHoldt BM (2012). STRUCTURE HARVESTER: a website and program for visualizing STRUCTURE output and implementing the Evanno method. Conserv. Gen. Resour..

[67] Jakobsson M, Rosenberg NA (2007). CLUMPP: a cluster matching and permutation program for dealing with label switching and multimodality in analysis of population structure. Bioinformatics.

[68] Ramasamy RK, Ramasamy S, Bindroo BB, Naik VG (2014). STRUCTURE PLOT: a program for drawing elegant STRUCTURE bar plots in user friendly interface. SpringerPlus.

[69] Peakall R, Smouse PE (2012). GenAlEx: genetic analysis in Excel. Population genetic software for teaching and research—an update. Bioinformatics.

[70] Vähä J-P, Erkinaro J, Niemelä E, Primmer CR (2007). Life-history and habitat features influence the within-river genetic structure of Atlantic salmon. Mol. Ecol..

[71] Kalinowski ST, Taper ML, Marshall TC (2007). Revising how the computer program CERVUS accommodates genotyping error increases success in paternity assignment. Mol. Ecol..

