# Supplementary material for: Population genetic analysis in old Montenegrin vineyards reveals ancient ways currently active to generate diversity in Vitis vinifera
Source: Sci Rep. 2020 Sep 14;10:15000. doi: 10.1038/s41598-020-71918-7 (PMC7490262; doi:10.1038/s41598-020-71918-7)
Supplement: Supplementary file 1 — Supplementary Figures [file 41598_2020_71918_MOESM1_ESM.pdf]

Population genetic analysis in old Montenegrin vineyards reveals ancient ways currently active to generate diversity in *Vitis vinifera*

Vesna Maraš[1], Javier Tello[2], Anita Gazivoda[1], Milena Mugoša[1], Mirko Perišić[1], Jovana Raičević[1], Nataša Štajner[3], Rafael Ocete[4], Vladan Božović[5], Tatjana Popović[6], Enrique García-Escudero[2], Miodrag Grbić[2,7,8], José Miguel Martínez-Zapater[2] and Javier Ibáñez[2,*]

[1]13 Jul Plantaže. Radomira Ivanovića br. 2, 8100 Podgorica, Montenegro.
[2] Departamento de Viticultura, Instituto de Ciencias de la Vid y del Vino (CSIC, UR, Gobierno de La Rioja). Ctra. de Burgos Km. 6, 26007 Logroño, Spain.
[3] Biotechnical Faculty, Agronomy Department, University of Ljubljana. Jamnikarjeva 101, 1000 Ljubljana, Slovenia.
[4] Laboratorio de Entomología Aplicada, Facultad de Biología, Universidad de Sevilla. Avenida de la Reina Mercedes s/n, 41012 Sevilla, Spain.
[5] Faculty for Food Technology, Food Safety and Ecology, University of Donja Gorica. Donja Gorica, 81000 Podgorica, Montenegro.
[6] Biotechnical Faculty, University of Montenegro. Mihaila Lalica 1, 81000 Podgorica, Montenegro.
[7] Department of Biology, University of Western Ontario. 1151 Richmond Street, N6A5B7 London, Canada.
[8] Faculty of Biology, University of Belgrade. Studentski trg. 16, Beograd 11000, Serbia.

[*] javier.ibanez@icvv.es

List of Supplementary Figures

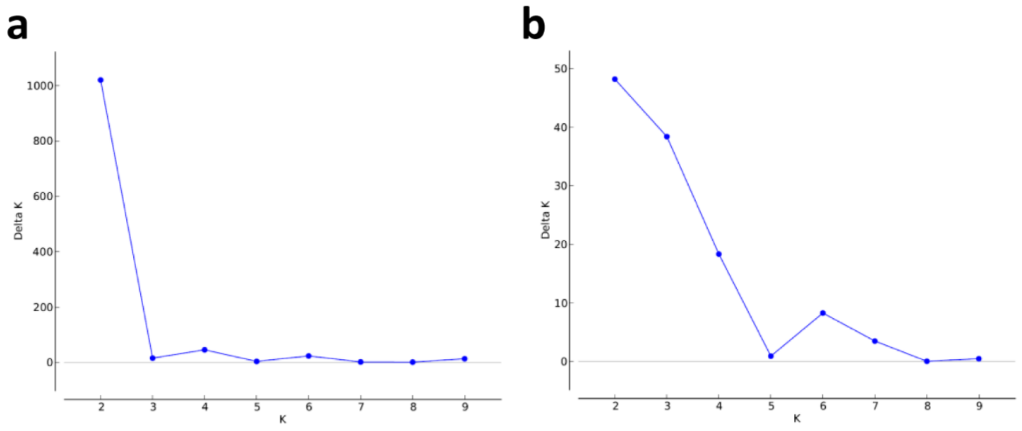

Supplementary Figure S1. Delta K plots obtained from STRUCTURE HARVESTER to set the most likely number of genetic groups present within 131 (A) or 91 (B) non-redundant grapevine genotypes identified in this study.

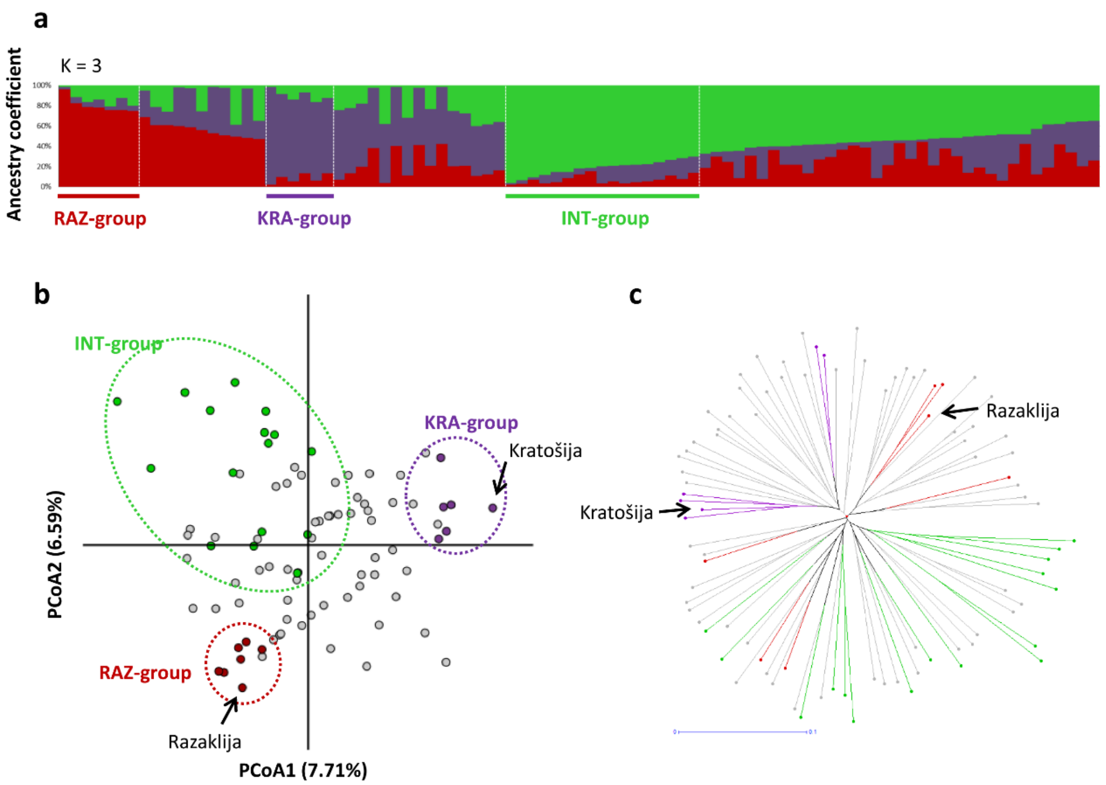

Supplementary Figure S2. Population structure analysis of 91 non-redundant cultivated varieties found in Montenegro**.** In A, STRUCTURE analysis also suggested the existence of three major genetic groups (RAZ-group, KRA-group and INT-group). Every non-redundant genotype is shown as a vertical line, with color segment lengths proportional to their inferred ancestry to the RAZ-group, KRA-group and INT-group (in red, purple and green, respectively). The number of genetic groups (K=3) was set considering the ΔK criterion[65]. Considering a critical ancestry coefficient of $q \geq 0.70$, 7, 6 and 17 genotypes were assigned to RAZ-group, KRA-group and INT-group, respectively (61 genotypes were considered as admixed). In B, a principal coordinate analysis (PCoA) obtained from a dissimilarity matrix calculated in DARwin from genetic data (194 SNPs) from the non-redundant cultivated genotypes is shown. The variance explained by the PCoA1 and PCoA2 is indicated as %. In C, the unweighted neighbor-joining (UwNJ) radiation tree obtained for the same dataset by means of DARwin is shown. In B and C, genotypes assigned to RAZ-group, KRA-group and INT-group are shown as red, purple and green dots, respectively (admixed genotypes are shown in grey).
